# P02.94. Acupuncture to decrease disparities in outcomes of pain treatment (ADDOPT): preliminary outcomes

**DOI:** 10.1186/1472-6882-12-S1-P150

**Published:** 2012-06-12

**Authors:** D McKee, B Kligler, A Blank, J Fletcher, F Biryukov, S George, W Casalaina, W Whitman, G Campos

**Affiliations:** 1 Albert Einstein College of Medicine, Bronx, USA; 2 Beth Israel Medical Center, Department of Integrative Medicine, New York City, USA; 3 Swedish Institute, New York City, USA; 4 Pacific College of Oriental Medicine, New York City, USA

## Purpose

Little is known about the integration of acupuncture for chronic pain in primary care settings, especially in low income and minority communities. The purpose of this ongoing NCCAM-funded study was to explore the feasibility and effectiveness of adding acupuncture to routine management in the primary care setting for diverse, low income chronic pain patients.

## Methods

Primary care physicians at four community health centers in the Bronx, NY referred adult patients with chronic pain due to osteoarthritis, neck or back pain for acupuncture. The acupuncture was offered weekly for up to 14 weeks at the primary care sites, delivered by student/preceptor teams from the Swedish Institute and the Pacific College of Oriental Medicine. Using a repeated measures design we evaluated changes in pain and function over a 6-month period as compared to baseline. Outcome measures were collected during a 6-week pre-treatment phase for baseline measures and then at 2, 4, 8, 12, and 16 weeks, with a final assessment at 6 months.

## Results

Regarding feasibility, we received over 400 referrals, reflecting a high level of interest in acupuncture services from both clinicians and patients. We also found that acceptability was high in that patients who began treatment tended to continue treatment, with a mean number of treatments per patient over 7. Regarding effectiveness, a preliminary analysis of completers to date found that pain severity and physical well-being were significantly improved from baseline at the six month follow-up. Mean BPI severity score decreased from 6.64 to 5.37 (p=.045, n=35). Mean SF-12 physical well-being score increased from 31.61 to 35.51 (p=.002, n=60).

## Conclusion

Acupuncture can be delivered in the primary care setting to chronic pain patients in an underserved area. A preliminary analysis shows that it can be effective in reducing pain and improving quality of life.

